# Excision of the Superior Maxilla

**Published:** 1885-04

**Authors:** 


### Excision of the Superior Maxilla.

Since it is well that we should know in what cases such an operation is justifiable, we note the conclusions of Dr. George A. Peters after reporting a case in the New York *Medical Journal*, January 17, 1885. This author considers the operation justifiable under the following conditions:

" Necrosis of the upper jaw, whether as the result of injury or disease in neighboring soft parts, syphilis, etc., requires operations sometimes even to the extent of removal of the entire jaw.

" Non-malignant tumors of the upper jaw, as fibroma and enchondroma, occasionally attain a size requiring removal of the entire bone.

" *Hyperostosis of the jaw*, which consists primarily in periosteal inflammation, leading to the deposit of new bone and the expansion and filling up of the original osseous structure, is unconnected with syphilis or struma, and appears to be beyond the control of remedies, and not infrequently reaches so great a size as to cause much deformity and justify the removal of the entire maxilla.

" It is in connection with malignant tumors of the upper jaw that the surgeon is often called upon to decide as to the propriety of removal.

"It is now well established by a large experience that the operation for cancer, even when so radical as the removal of the entire jaw, does not result in a cure, the disease being almost certain to recur. Nevertheless, the possibility of a non-recurrence, and the positive temporary relief afforded, in connection with the moral effect of the operation, will justify surgical interference."

